# Obesity, preoperative weight loss, and telemedicine before total joint arthroplasty: a review

**DOI:** 10.1186/s42836-021-00102-7

**Published:** 2022-01-04

**Authors:** Michael W. Seward, Antonia F. Chen

**Affiliations:** 1 Mayo Clinic, Department of Orthopedic Surgery, 200 1st St SW, Rochester, MN 55905 USA; 2 Brigham and Women’s Hospital, Department of Orthopaedic Surgery, 75 Francis Street, Boston, MA 02115 USA

**Keywords:** Arthroplasty, Dietitian, Knee osteoarthritis, Mobile applications, Obesity, Osteoarthritis, hip, Preoperative period, Smartphone, Telemedicine, Weight loss

## Abstract

The preoperative period prior to elective total joint arthroplasty (TJA) is a critical time for lifestyle interventions since a scheduled surgery may help motivate patients to lose weight. Weight loss may reduce complications associated with obesity following TJA and enable patients with severe obesity (body mass index [BMI] > 40 kg/m^2^) to become eligible for TJA, as many institutions use a 40 kg/m^2^ cut-off for offering surgery. A comprehensive review was conducted to (1) provide background on complications associated with obesity following TJA, (2) synthesize prior research on the success rate of patients losing weight after being denied TJA for severe obesity, (3) discuss bariatric surgery before TJA, and (4) propose mobile health telemedicine weight loss interventions as potential weight loss methods for patients preoperatively.

It is well established that obesity increases complications associated with TJA. In total knee arthroplasty (TKA), obesity increases operative time, length of stay, and hospitalization costs as well as the risk of deep infection, revision, and component malpositioning. Obesity may have an even larger impact on complications associated with total hip arthroplasty (THA), including wound complications and deep infection. Obesity also increases the risk of hip dislocation, aseptic loosening, and venous thromboembolism after THA.

Synthesis of the only two studies (*n* = 417), to our knowledge, that followed patients denied TJA for severe obesity demonstrated that only 7% successfully reduced their BMI below 40 kg/m^2^ via lifestyle modifications and ultimately underwent TJA. Unfortunately, bariatric surgery may only increase certain post-TKA complications including death, pneumonia, and implant failure, and there is limited research on preoperative weight loss via lifestyle modification. A review of short-term mobile health weight loss interventions that combined personalized counseling with self-monitoring via a smartphone app found about 5 kg of weight loss over 3-6 months. Patients with severe obesity have more weight to lose and may have additional motivation to do so before TJA, so weight loss results may differ by patient population. Research is needed to determine whether preoperative mobile health interventions can help patients become eligible for TJA and produce clinically significant weight loss sufficient to improve postoperative outcomes.

## Introduction

Patients should weigh the risks and benefits of undergoing elective surgery, which often involves considering comorbidities that may increase the risk of procedures [1, 2]. Total joint arthroplasty (TJA) is a common elective surgery that can be scheduled to give patients several months to prepare for surgery and make lifestyle changes to minimize the risk of comorbidities [3]. Mitigating risk factors before surgery can improve patient outcomes, and bundled payment models may offer additional clinical and financial incentives to optimize risk factors before surgery [4]. The purposes of this review are to: (1) provide background on complications associated with obesity following TJA, (2) synthesize prior research on the success rate of patients losing weight after being denied TJA for severe obesity (body mass index [BMI] > 40 kg/m^2^), (3) aggregate the latest literature examining bariatric surgery before TJA, and (4) review mobile health telemedicine weight loss interventions as potential weight loss methods for patients preoperatively.

## Obesity

The risk of knee osteoarthritis is four times higher for men with obesity and five times higher for women with obesity [5]. The risk of undergoing total knee arthroplasty (TKA) in patients with a body mass index (BMI) over 40 kg/m^2^ is similarly quadrupled for patients diagnosed with knee osteoarthritis under the age 68 years, and doubled for patients diagnosed at age 68 years or older compared to patients of normal weight with osteoarthritis [6]. Data from the Canadian Joint Replacement Registry were even more concerning, showing a 33 and nine times higher risk of TKA and total hip arthroplasty (THA), respectively, for patients with severe obesity [7].

In TKA, obesity increases the risk of deep infection and rates of revision [8], operative time [9, 10], component malpositioning [11], length of stay, and both hospitalization and total 90-day costs [12]. Severe obesity among TKA patients is associated with even longer length of stay [13–15], lower absolute physical functional improvement [16], increased 30-day mortality [13, 17], discharge to a rehabilitation facility [13–16], and deep vein thrombosis and pulmonary embolism [18]. Patients undergoing TJA with a BMI over 45 kg/m^2^ have over eight times higher risk of in-hospital complications [19].

Some comorbidities, such as obesity and diabetes, were thought to have similar effects on the risks associated with both THA and TKA [20], so previous analyses of complications often combined risks or were limited to risks associated with TKA. A large database study by DeMik et al in 2018 was the first to investigate if obesity conferred higher risks for either of the two procedures [21]. The study included 64,648 patients who underwent THA and 97,137 patients who underwent TKA and found that the impact of obesity on postoperative complications was even larger for THA than TKA. Among patients with severe obesity, the odds of total complications (odds ratio [OR], 1.325; 95% CI, 1.159–1.515), wound complications (OR, 2.092; 95% CI, 1.687–2.594), deep infection (OR, 3.811; 95% CI, 2.369–6.130), and reoperation (OR, 2.249; 95% CI, 1.832–2.762) were higher after THA than TKA. When analyzing THA alone, obesity increased the likelihood of hip dislocation, aseptic loosening, infection, and venous thromboembolism [22].

**Fig. 1:**
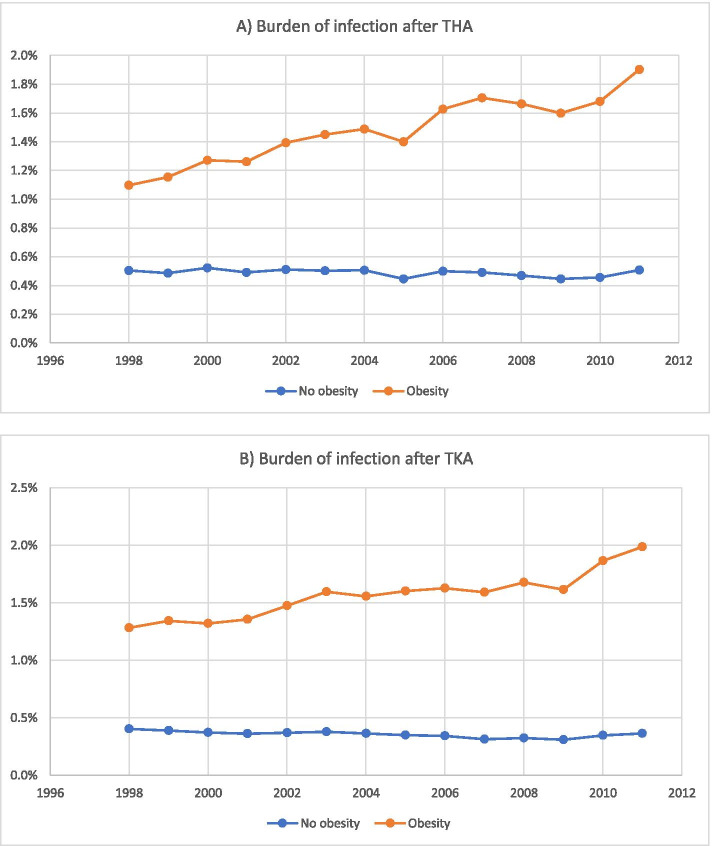
Increased infection burden (infected procedures/total procedures) in patients with obesity after (A) total hip arthroplasty (THA) and (B) total knee arthroplasty (TKA) from 1998 to 2011 in the United States. Recreated from George et al. [24]

The effect of obesity on TJA complications deserves special attention given the ongoing obesity epidemic in the United States. Fehring et al found the prevalence of obesity among patients undergoing TJA increased from 30.4% in 1990 to 52.1% in 2005 at a single institution [23]. A later national analysis by George et al found the prevalence of obesity increased from 39% in 1998 to 52% in 2011 (increase of 1.05%/year) among patients undergoing primary THA and increased from 57% in 1998 to 70% in 2011 (increase of 0.97%/year) among patients undergoing primary TKA [24]. The report found similar trends for obesity with respect to TJA revisions and infection burden (Fig. 1) [24].

## Success rate of patients reducing BMI prior to TJA without bariatric surgery

An upcoming TJA may motivate patients with severe obesity to lose weight, both because preoperative weight loss may improve outcomes following TJA and because it may help them become eligible for TJA if they have a BMI above a common cutoff of 40 kg/m^2^ [25]. Many practices adopted this cut-off after the American Association of Hip and Knee Surgeons (AAHKS) recommended in 2013 delaying TJA above this BMI because complication profiles of these patients may outweigh the functional benefits of surgery [26].

There are two recent observational studies that followed patients initially denied TJA for severe obesity who were told to lose weight to become eligible for surgery. The low success rate of these patients reducing their BMI after denial can provide useful context when evaluating preoperative weight loss interventions.

The first observational study by Shapiro et al in 2020 analyzed patients with a minimum 2 year follow-up who were denied TJA due to severe obesity at the University of North Carolina at Chapel Hill [27]. At that institution, patients with a BMI over 40 kg/m^2^ were initially denied TJA unless they lost greater than two-thirds of the weight necessary to achieve a BMI of 40 kg/m^2^. For example, a patient with a starting BMI of 46 kg/m^2^ would be offered TJA if they reached a BMI of 42 kg/m^2^. Of 125 patients initially denied TJA due to severe obesity, 19.2% (24 of 125) ultimately met “target” weight and underwent TJA, while 80.8% (101 of 125) failed to reach “target” weight and were not offered TJA. Among those who did not reach “target” weight, 21.6% (27 of the initial 125) sought second opinions at another institution, and 6.4% (8 of the initial 125) ultimately had TJA with another institution. Among patients initially denied TKA, 10.1% (10 of 99) reached a BMI at or below 40 kg/m^2^ via lifestyle modifications only and ultimately underwent TKA (Table 1). Among patients initially denied THA, 3.4% (1 of 29) reached a BMI at or below 40 kg/m^2^ and ultimately underwent THA, though the paper did not specify if that individual did so via lifestyle modifications or bariatric surgery. If that individual lost weight via lifestyle modifications, the overall success rate of achieving a BMI under 40 kg/m^2^ without bariatric surgery was 8.6% (11 of 128).

**Table 1:** Relevant prior observational studies investigating success rates of patients with severe obesity initially denied total knee arthroplasty (TKA) or total hip arthroplasty (THA) reaching a body mass index (BMI) < 40 kg/m^2^ without bariatric surgery

	Achieved BMI < 40 before TKA	Achieved BMI < 40 before THA	Achieved BMI < 40 before TJA
Shapiro study (*n* = 128) [27]	10.1%	0.0% or 3.4%*	7.8% or 8.6%*
Springer study (*n* = 289) [28]	4.2%	14.7%	6.9%
Overall (*n* = 417)	6.1%	10.6% or 11.5%*	7.2% or 7.4%*

*TKA total knee arthroplasty; THA total hip arthroplasty; TJA total joint arthroplasty*

*The paper by Shapiro et al did not specify if the one individual who achieved BMI < 40 before THA did so via lifestyle modifications or bariatric surgery so both rates are listed.

A second study by Springer et al in 2019 prospectively followed 289 patients for 2 years who otherwise would have qualified for TJA but were denied due to having a BMI over 40 kg/m^2^ [28]. All patients were given phone numbers, but not referred, for two local bariatric clinics offering either medical or surgical weight loss. Of these patients, 28.4% (82 of 289) were completely disengaged and lost to follow-up, and only 23.2% (67 of 289) scheduled and attended an appointment at the bariatric clinics, highlighting some of the challenges of engaging this population of patients. The study searched three electronic medical record systems that covered nearly 90% of healthcare visits in the study’s region to investigate if denied patients lost to follow-up went to providers at other institutions, so the authors felt the vast majority, if not all these patients, lost to follow-up did not undergo TJA elsewhere. Of the initial 289 patients denied TJA, 6.9% (20 of 289) achieved a BMI < 40 kg/m^2^ without bariatric surgery and underwent TJA (Table 1). This included 14.7% (11 of 75) of those initially denied THA and 4.2% (9 of 214) initially denied TKA.

## Bariatric weight loss surgery before TJA

Bariatric surgery is sometimes recommended to patients with severe obesity to help lose weight before TJA, and can result in mean total weight loss exceeding 31% [29]. A 2016 systematic review and meta-analysis of five studies compared 657 patients who had obesity and underwent bariatric surgery before TJA to 22,691 patients who had obesity but did not undergo bariatric surgery before TJA [30]. The mean BMI at TJA was 36.1 kg/m^2^ in the bariatric surgery group, and 42.9 kg/m^2^ in the non-bariatric surgery group. There were no significant differences in superficial or deep wound infections, deep vein thrombosis, pulmonary embolism, joint revision, mortality, in-patient re-admission, postoperative blood transfusion, or complications within the first 90 postoperative days. Although there were lower risks of overall medical complications collectively assessed (relative risk [RR], 0.54; 95% CI, 0.39–0.74) and overall postoperative wound infections (RR, 0.36; 95% CI, 0.15–0.90) in patients undergoing bariatric surgery before TJA, the authors concluded that bariatric surgery may not be helpful for reducing the risk of post-TJA complications. However, all the reviewed studies were retrospective in design and not randomized, and none of the studies identified the type of bariatric surgery procedure used (e.g., banding, sleeve, Roux-en-Y bypasses, etc.).

A more recent (2018) large retrospective study of Medicare patients undergoing THA (*n* = 47,895) and TKA (*n* = 86,609) found mixed results for bariatric surgery before TJA [31]. The paper did not report the mean BMI of patients at the time of TJA or the success rate of bariatric surgery. Compared to patients with common metabolic conditions but without bariatric surgery before THA, patients who had bariatric surgery had 12.8 (*P* = 0.009), 10.1 (*P* = 0.017), and 7.7 (*P* = 0.038) times greater risk of periprosthetic joint infection at 0.5, 1, and 2 years, respectively, but no significant difference in risk of revision. Conversely, patients who had bariatric surgery before TKA had 4.3 (*P* = 0.003), 3.6 (*P* = 0.004), and 3.4 (*P* = 0.003) times greater risk of revision at one, two, and five years, respectively, but no significant difference in risk of periprosthetic joint infection. Again, the study did not distinguish between the types of bariatric procedures used before TJA.

Another retrospective study of 2.7 million Medicare patients, including 25,852 patients who underwent bariatric surgery before TKA, compared outcomes based on the type of bariatric procedure performed over an 11-year period [32]. Of the bariatric surgery techniques, Roux-en-Y bypasses produce the greatest average weight loss [29], and were therefore initially hypothesized to have the greatest potential to reduce risks associated with TKA in patients with severe obesity. Indeed, patients undergoing Roux-en-Y bypasses before TKA had lower risks of post-TKA periprosthetic infection (HR, 0.41), renal failure (HR, 0.71), revision (HR, 0.41), and wound dehiscence (HR, 0.67) compared to patients with severe obesity not undergoing bariatric surgery. However, patients undergoing gastric bypass before TKA had a notably higher risk of death (HR, 1.47) and pneumonia (HR, 1.68), while patients undergoing sleeve gastrectomy had higher risk of implant failure (HR, 1.58). Overall, the authors concluded that bariatric surgery is not sufficient to normalize risks after TKA and may actually increase certain post-TKA complication risks depending on the type of bariatric procedure used.

## Mobile health (mHealth) telemedicine interventions for weight loss

Given the lack of consistent evidence supporting bariatric surgery before TJA, practices have also considered behavioral and dietary weight loss interventions. In fact, positive preoperative lifestyle factors, including non-smoking status, increased physical activity, and normal body mass index (BMI), are associated with improved postoperative outcomes [33–35]. However, time pressure, fiscal constraints, and limited provider experience encouraging lifestyle changes may hamper effective counseling during the preoperative period [36, 37]. Most health professionals have difficulty providing practical lifestyle advice to patients [38], and one study found little differences in the diet-related knowledge between health professionals and the general public [39]. Digital technologies, including mobile health (mHealth), have recently been proposed as a potential solution to the barriers of preoperative lifestyle counseling [2]. While the COVID-19 pandemic has sparked interest in telerehabilitation, or mHealth interventions that optimize patients before surgery [40, 41], there is little research on preoperative mHealth weight loss interventions.

Digital technologies make up the rapidly growing field of mHealth. Mobile health technologies can be divided into five general categories: (1) a mobile app that uses smartphones; (2) web-based tools; (3) text messaging; (4) portable monitoring device/personal digital assistant (PDA), which usually collects patient data over a wireless connection to monitor patients’ physiological status; and (5) pedometers or wearable trackers [42]. These technologies can offer direct, affordable health services that may improve patient adherence to health care providers’ advice, patient-provider communication, and facilitate behavior change [43, 44]. There are no prior studies of mobile health interventions for preoperative weight loss before total joint arthroplasty, therefore, it is difficult to estimate the cost and financial feasibility of implementing such strategies at orthopaedic surgery clinics. The cost of these five mobile health services varies even within each general category. For example, some weight loss mobile apps and web-based tools (categories 1 and 2) may include free nutritional guidance and calorie counting tools, while others may additionally offer live video calls with registered dietitians and charge $35 per 25-min session [45]. Similarly, PDA’s and wearable trackers (categories 4 and 5) vary considerably by included features and price; trackers from popular consumer brands like Fitbit range from about $50 to $250 each [46]. However, recent research suggests that remote telephonic health coaching may be a cost-effective alternative to promoting physical activity in patients who underwent TKA, which could potentially contribute to preoperative weight loss [47]. More affordable obesity treatment may be critical for some patients because most insurance plans lack or only provide limited coverage for intensive weight loss counseling. As a result, patients are often required to pay for nutrition services out of pocket [48]. A study in 2018 of insurance coverage for obesity treatment services found that Medicaid programs in only 21 states included any nutritional counseling [49].

Nutrition counseling via mobile health may be particularly effective in orthopedic clinics. A study of mobile health literacy and mobile device use among older patients over age 50 years (59% were over age 65 years) seen in orthopedic clinics found that 81% owned at least one mobile device and there was significant adoption of mobile technology among older adults. Patients aged 65 to 74 years had similar mobile health literacy to those aged 50 to 64 years, and the authors concluded that interactive mHealth interventions could improve patient engagement and musculoskeletal health management for patients with orthopedic problems [50]. However, there is no research on mobile health weight loss interventions before orthopedic surgery. A review of the overall mobile health weight loss literature may provide insights into how to design an effective mobile health preoperative intervention.

A 2019 systematic review and meta-analysis involving 2318 subjects with obesity among 20 randomized controlled trial (RCTs) of mHealth interventions specifically using mobile phones found a weighted mean difference of − 2.25 kg (95% CI; − 3.34, − 1.16) at three to four months, and − 2.66 kg (95% CI; − 3.94, − 1.38) at 6 months compared to groups not receiving a mobile device intervention. There was a high level of study heterogeneity and a statistically insignificant BMI reduction of − 1.10 kg/m^2^ (95% CI; − 2.79, 0.59) for studies measuring outcomes at 3 months, but low study heterogeneity and a statistically significant BMI reduction of − 0.67 kg/m^2^ (95% CI; − 0.71, − 0.63) at 6 months [51].

Importantly, the 2019 mHealth review analyzed a variety of mobile phone interventions, but just four of the 20 identified RCTs provided feedback or coaching based on monitored results (e.g., counseling on weight or behaviors recorded by mobile devices). Two of the four mHealth RCTs provided only limited coaching via automated text messages [52, 53]. The study by Sidhu et al included subjects with a mean BMI of 34.3 kg/m^2^ and found no difference in weight loss maintenance using a text messaging intervention [52]. The study by Lin et al included participants with a mean BMI of 28 kg/m^2^ and found the intervention group lost about 1.8 kg more than the control group [53]. The third study was a pilot RCT by Allen et al that combined face-to-face personalized counseling with self-monitoring via a smartphone app. The study was not powered to detect statistically significant differences in weight, though mean weight loss at 6 months in the group receiving counseling plus a self-monitoring smartphone app was − 5.4 kg. There was no meaningful weight loss in the group receiving the self-monitoring app alone, suggesting that having some degree of personal contact such as face-to-face or remote counseling was crucial for weight loss [54]. The fourth study was a 12-month RCT by Spring et al that compared a PDA plus telephone coaching group (+Mobile) to a standard care (Standard) group receiving a group weight loss program. At 3 months, the +Mobile group lost an average of 9.7 pounds (95% CI; 6.0, 13.5) compared to 1.9 pounds (95% CI; 0.1, 4.0) in the Standard group [55]. These studies by Allen et al (− 5.4 kg over 6 months) and Spring et al (− 4.4 kg over 3 months) may give a reasonable estimate of weight loss to be expected from a short-term mHealth weight loss intervention incorporating personalized counseling. However, the four RCTs combining mobile devices and some form of coaching included subjects with average BMIs of 28.2 to 36.3 kg/m^2^, limiting generalizability to subjects with severe obesity [52–55]. More research is needed to determine whether potentially greater weight loss might be expected from patients with severe obesity who have more weight to lose. For example, a recent review of preoperative weight loss interventions before total joint arthroplasty for patients with obesity identified five studies. None of the five studies included mobile health interventions, but preoperative weight loss ranged from 5.0 to 32.5 kg [56].

There is limited research on the amount of preoperative weight loss necessary to optimize clinical outcomes following TJA [57]. In a retrospective review of 203 patients at one institution with BMI over 40 kg/m^2^ who lost weight before TKA, losing at least 20 pounds before TKA was associated with fewer discharges to a facility and shorter length of stay, but losing only five or ten pounds had no effect [58]. A 20-pound preoperative weight loss exceeds the 5 kg weight loss found in prior mobile health interventions [54, 55], though patients with severe obesity have more weight to lose and may have extra motivation to lose weight before TJA. Previous mobile health interventions with overweight pregnant women found patients had additional impetus to make lifestyle changes due to the upcoming delivery [59], so an upcoming surgery may be similarly motivating. More research is needed to determine whether preoperative mobile health interventions can produce clinically significant weight loss sufficient to improve outcomes after TJA. However, even more modest weight loss may help patients become eligible for TJA and mobile health interventions may increase access to obesity treatment given the considerable evidence that weight bias and stigma cause avoidance of care and poor treatment adherence [60].

## Conclusions

A motivating event like an upcoming elective TJA may be a unique opportunity for lifestyle interventions. Preoperative weight loss may both improve postoperative clinical outcomes and help more patients with severe obesity to become eligible for TJA. In this review, we synthesized the only two studies (*n* = 417) to our knowledge that followed patients denied TJA for severe obesity [27, 28]. Only about 7% of these patients reduced their BMI below 40 kg/m^2^ via lifestyle modifications alone, and bariatric surgery may increase certain post-TKA complications, including implant failure, pneumonia, and death [32]. Unfortunately, effective nutritional counseling by clinicians and surgeons is limited by constraints on time, financial support, experience, and weight stigma [36, 37, 60]. Short-term mobile health weight loss interventions that combine personalized counseling with self-monitoring via a smartphone app can produce about 5 kg of weight loss over three to six months [54, 55]. Future studies should assess whether preoperative mobile health weight loss interventions increase access to obesity treatment and help patients lose weight prior to TJA [61]. Counterintuitively, patients undergoing TJA with severe obesity are more likely to have malnutrition than patients without severe obesity, [62] such that preoperative weight loss studies should also investigate changes in blood serological markers of malnutrition to ensure that weight loss does not exacerbate malnutrition preoperatively [63]. These interventions should be evaluated with the context that only 7% of patients with severe obesity needing TJA typically achieve BMIs below 40 kg/m^2^. Additional research should determine the amount of clinically significant preoperative weight loss to ensure these interventions improve outcomes after TJA [56]. Nonetheless, preoperative mobile health interventions may offer patients with severe obesity an additional weight loss method to become eligible for TJA.

## Data Availability

Not applicable.

## Abbreviations

BMI: Body mass index
EMR: Electronic medical record
HR: Hazard ratio
mHealth: Mobile health
OR: Odds ratio
PDA: Personal digital assistant
RCT: Randomized controlled trial
RR: Relative risk
THA: Total hip arthroplasty
TJA: Total joint arthroplasty
TKA: Total knee arthroplasty
